# Prostate Cancer Burden at the Uganda Cancer Institute

**DOI:** 10.1200/JGO.2015.001040

**Published:** 2016-02-17

**Authors:** Fred Okuku, Jackson Orem, George Holoya, Chris De Boer, Cheryl L. Thompson, Matthew M. Cooney

**Affiliations:** **Fred Okuku, Jackson Orem,** and **George Holoya,** Uganda Cancer Institute, Kampala, Uganda; **Chris De Boer,** University of Iowa Carver College of Medicine, Iowa City, IA; and **Cheryl L. Thompson** and **Matthew M. Cooney,** Case Western Reserve University, Cleveland, OH.

## Abstract

**Purpose:**

In Uganda, the incidence of prostate cancer is increasing at a rate of 5.2% annually. Data describing presentation and outcomes for patients with prostate cancer are lacking.

**Methods:**

A retrospective review of medical records for men with histologically confirmed prostate cancer at the Uganda Cancer Institute (UCI) from January 1 to December 17, 2012, was performed.

**Results:**

Our sample included 182 men whose mean age was 69.5 years (standard deviation, 9.0 years). Patients who presented to the UCI had lower urinary tract symptoms (73%; n = 131), bone pain (18%; n = 32), increased prostate-specific antigen (PSA; 3%; n = 5), and other symptoms (6%; n = 11). Median baseline PSA was 91.3 ng/mL (interquartile range, 19.5-311.3 ng/mL), and 51.1% of the patients (n = 92) had a PSA value above 100 ng/mL. Gleason score was 9 or 10 in 66.7% of the patients (n = 120). Ninety percent (n = 136) had stage IV disease, and metastatic sites included bone (73%; n = 102), viscera (21%; n = 29), and lymph nodes (4%; n = 5). Spinal cord compression occurred in 30.9% (n = 55), and 5.6% (n = 10) experienced a fracture. A total of 14.9% (n = 27) underwent prostatectomy, and 17.7% (n = 32) received radiotherapy. Gonadotropin-releasing hormone agonist was given to 45.3% (n = 82), 29.2% (n = 53) received diethylstilbestrol, and 26% (n = 47) underwent orchiectomy. Chemotherapy was administered to 21.6% (n = 39), and 52.5% (n = 95) received bisphosphonates. During the 12 months of study, 23.8% of the men (n = 43) died, and 54.4% (n = 98) were lost to follow-up.

**Conclusion:**

UCI patients commonly present with high PSA, aggressive Gleason scores, and stage IV disease. The primary treatments are hormonal manipulation and chemotherapy. Almost 25% of patients succumb within a year of presentation, and a large number of patients are lost to follow-up.

## INTRODUCTION

Prostate cancer is the most common cancer among men in Africa and the third most common cancer overall with 59,500 incident cases per year (16.4% of all cancer in men).^1-3^ In sub-Saharan Africa, the relative burden is higher, with prostate cancer representing 20.3% of all cases of cancer in men.^3^ Incidence rates are highest in Zimbabwe and Uganda, with incidences of 38.1 and 37.1 per 100,000, respectively, which is still far below the rate of black American men at 172.8 per 100,000.^4^ However, these estimates may be low because another source estimates incidence of prostate cancer in Uganda at 65.0 per 100,000.^3^ The lack of cancer registries and regional estimates makes it difficult to accurately estimate incidences across different countries, and underreporting of prostate cancer is suspected.^4^ Mortality from prostate cancer remains high as well; the mortality:incidence ratio is 71%.^3^ Despite these numbers, research focusing on prostate cancer in Africa is lacking, and many questions remain with respect to patient characteristics, risk factors, and outcomes.

In addition to increasing incidence and mortality attributed to greater case detection, aging, and changing lifestyles, a clear association has been made between higher incidence and individuals of African descent. Population-based studies have shown that African American men have among the highest incidence rates of prostate cancer in the world.^5^ In addition, they are more likely to experience worse prognoses and lower survival rates.^6,7^ Genetics is suspected to be linked to these observations, and a genetic link between Africans with prostate cancer and African American men with prostate cancer has been proposed.^8^

Despite these findings, there is a lack of data surrounding prostate cancer in Africa, and there are few population-based studies that have analyzed genetic variants, patient characteristics, risk factors, and outcomes for men with prostate cancer. In South Africa, genotyping found that a cohort of patients with prostate cancer shared no previously defined risk alleles with European cohorts and that patients were more likely to have a family history of cancer, diabetes, erectile dysfunction, balding, frequent aspirin use, and increased prostate-specific antigen (PSA) levels in a case-control analysis.^9^ Population-based studies in Ghana and Nigeria have also found significant incidence and mortality from prostate cancer in those countries.^10^ In Nigeria, some studies suggest that prostate cancer incidence could be as high as that among black men in the United States.^11^ In Uganda, it is estimated that incidence is increasing by 5.2% annually, making it the most rapidly increasing cancer in the country.^12^ These results have provided initial data on prostate cancer in Africa, but many questions remain.

In response to this gap in understanding, a retrospective review of medical records among patients with prostate cancer was conducted at the Uganda Cancer Institute (UCI) in Kampala, Uganda, on new patients who presented for evaluation from January 1 to December 17, 2012. The aim of our study was to describe patient characteristics, treatments given, and survival of patients referred to the UCI.

## METHODS

This study was a retrospective review of the medical records of new patients with prostate cancer who received treatment at the UCI from January 1 through December 17, 2012. All patients had histologic confirmation of a diagnosis of prostate cancer before being referred to the UCI and were at least 18 years old. Data from medical records were abstracted to obtain patient demographic characteristics, presenting symptoms, clinical stage, treatment exposures, and overall survival. Total time on study for each participant was defined as the date of pathologic diagnosis until the last follow-up in the 2012 calendar year or death. This study was approved by the Mulago Hospital Ethics Committee and was supported by the UCI.

Means with standard deviations, medians with interquartile ranges, averages, percentages, and incidence rates were used to describe patient characteristics. Median survival times and hazard ratios were obtained from Cox regression models. Data were stored with Epi Info and analyzed with STATA/SE version 12.0 (STATA, College Station, TX).

## RESULTS

Because of concerns about disease progression, men were referred from outside institutions to the UCI for further evaluation of their prostate cancer. Of these, 182 men enrolled had a median age of 70 years (interquartile range [IQR], 64 to 75 years). Before being referred to the UCI, a total of 14.9% patients (n = 27) underwent a radical prostatectomy for curative intent. Their pathologic Gleason score was 9 or 10 in 66.7% (n = 120), 7 to 8 in 23.4% (n = 44), and 6 or lower in 10% (n = 18). Although 17.7% of patients (n = 32) received palliative radiotherapy, none received definitive radiotherapy for curative intent for their prostate cancer.

Upon initial evaluation at the UCI, the primary complaints were lower urinary tract symptoms (73%; n = 131), bone pain (18%; n = 32), increased PSA (3%; n = 5), and other causes (6%; n = 11). Initial PSA readings at the UCI included a median PSA of 91.3 ng/mL (IQR, 19.5 to 311.3 ng/mL), and of patients with initial PSA readings, 51.1% (n = 92) had a PSA value above 100 ng/mL (Table 1). Cardiovascular disease (47.5%; n = 84) was the most common comorbid condition in the patient population. Of the 177 patients who were tested for HIV, only one patient (0.6%) tested positive.

**Table 1:**
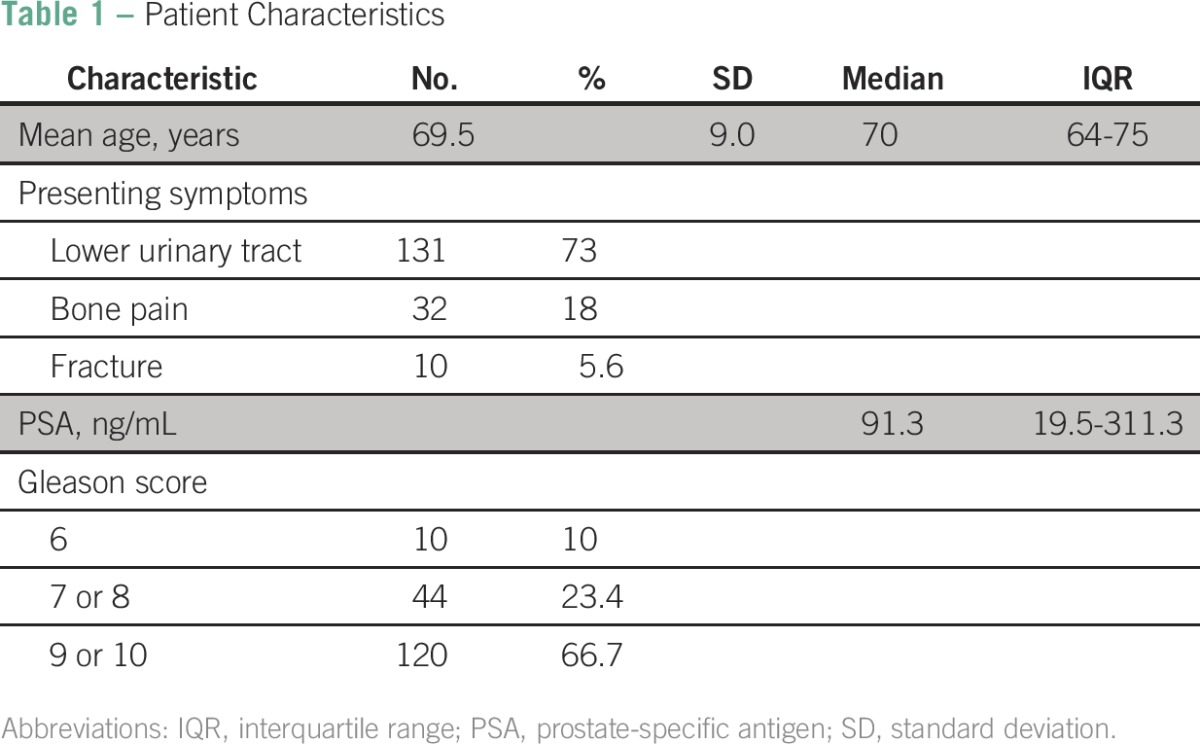
Patient Characteristics

All patients were offered staging by physical examination, bone scan, and computed tomography scans. However, because of the lack of financial resources, 14 men were not able to obtain imaging studies. Upon presentation, 90% of patients (n = 136) had stage IV disease, 6.5% (n = 11) had stage III, 11.9% (n = 20) had stage II, and 0.6% (n = 1) had stage I. Common sites of metastases included bone (73%; n = 102), viscera (21%; n = 29), and lymph nodes (4%; n = 5). Complications involving the skeleton included spinal cord compression in 30.9% of patients (n = 55), and 5.6% (n = 10) experienced a fracture (Table 2).

**Table 2:**
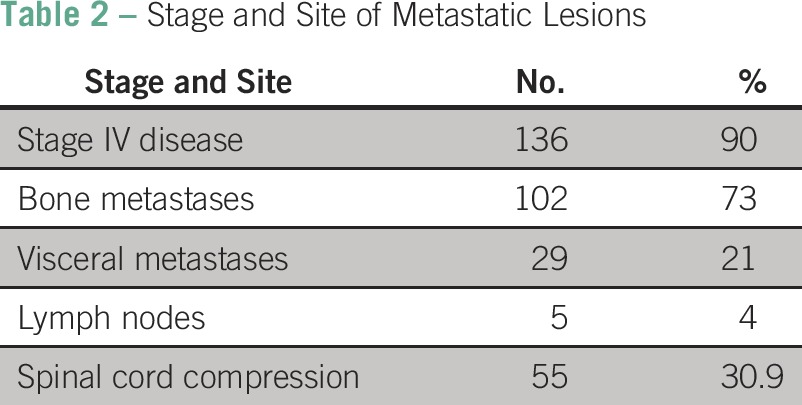
Stage and Site of Metastatic Lesions

Treatment given at the UCI included at least one dose of gonadotropin-releasing hormone (GnRH) agonist to 45% of patients (n = 82), 29% (n = 53) received diethylstilbestrol (DES), and 26% (n = 47) underwent bilateral orchiectomy. Taxane-based chemotherapy was administered to 21.6% of patients (n = 39), and 52.5% (n = 95) received bisphosphonate therapy (Table 3).

**Table 3:**
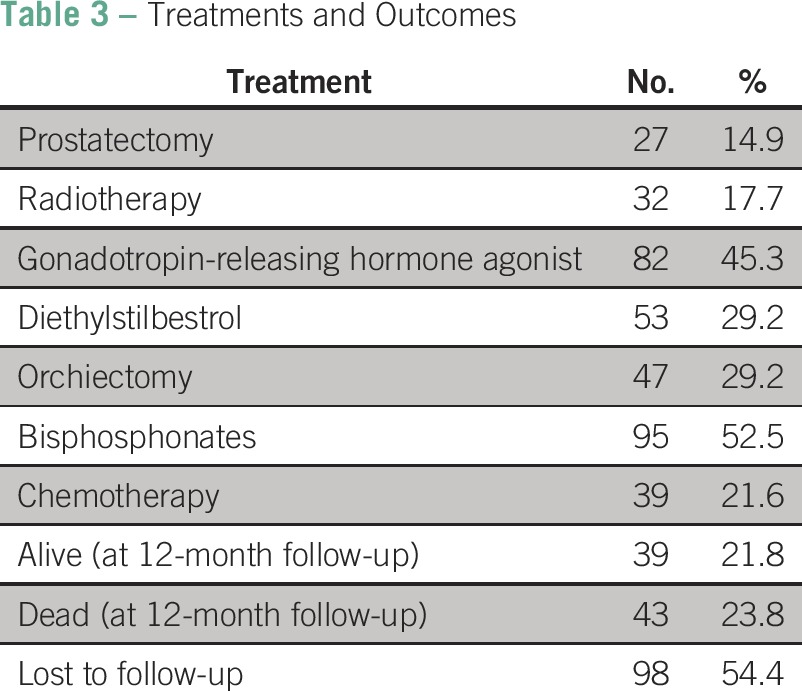
Treatments and Outcomes

Between January 1 and December 17, 2012, 23.8% of men (n = 43) died and 54.4% (n = 98) were lost to follow-up. From the time of pathologic diagnosis, the median survival time was 3,659 days for those with stage IV disease, 4,063 days for stage III, and 4,504 days for stage II. The patient with stage I was lost to follow-up before the end of data collection.

## DISCUSSION

Prostate cancer is an increasingly important cancer in Africa with respect to morbidity and mortality because incidence rates are increasing, particularly within Uganda. Unfortunately, there are few studies of prostate cancer in sub-Saharan Africa. In response to this gap in understanding, this study described patient presentation and treatments given among men with prostate cancer at the UCI in Kampala, Uganda.

Patients in this study were diagnosed with prostate cancer and then referred to the UCI. The time of pathologic diagnosis to the median survival time was more than 3,600 days. However, by the time the patients are referred to the UCI, approximately 90% have stage IV disease. It is thus not surprising that there is a high morbidity and mortality rate for men with prostate cancer during their first year of evaluation at the UCI. Almost 33% of patients experience cord compression, and almost 25% of patients succumb within a year of presentation. Therefore, the majority of prostate cancer treatment in Uganda is palliative with radical prostatectomy being uncommon. In the future, interventions need to be launched to increase earlier referral to the UCI for patients with prostate cancer.

Access to safe and reliable radiotherapy treatments in sub-Saharan Africa is a major obstacle. There is limited availability to radiotherapy in low-income countries because of their expense, lack of technical training and/or professional staff, lower ability to maintain and repair the technology, and a lack of sustained electrical supply. In Uganda, there is one cobalt therapy unit that is more than 20 years old to serve more than 30 million people. When men are diagnosed with limited-stage prostate cancer, this cobalt unit is not optimal for their treatment because of concerns about toxicity. Thus, in this report, less than 20% of men received radiotherapy, and it was all in the palliative setting.

Hormonal manipulation, which includes lowering the testosterone to castrate levels, is the mainstay of palliative therapy for patients with prostate cancer. In many low-income countries, access to a stable and high-quality supply chain for cancer treatments can be a challenge. In this study, because of an inadequate and inconsistent supply of GnRH analogs, less than half the patients received GnRH; DES was substituted instead. Prescribing DES is suboptimal because of the increased risk of cardiovascular complications, stroke, and blood clots. In addition, DES prescriptions need to be continuously renewed, which makes it a challenge for the high numbers of patients who were lost to follow-up.

Although it is inexpensive and permanent, bilateral orchiectomy is uncommon. It is unclear why the orchiectomy rate is so low. Hypotheses include lack of willingness to undergo the procedure, scarcity of urologists to perform the surgery, or possibly inability to secure time in the surgical theater. Future projects need to assess patients’ attitudes, availability of surgeons, and availability of surgical theater space to determine how to increase the orchiectomy rate for palliation of prostate cancer.

The UCI, which is the main referral source for more than 30 million Ugandans, also treats patients from neighboring Sudan, Congo, Burundi, Rwanda, and Kenya. Because health care in low-income countries is often fragmented, this makes continuity of care difficult, and a major limitation of this study was that more than 50% of patients were lost to follow-up. Designing interventions to improve treatment adherence, such as using appointment reminders via mobile phones, is an absolute necessity in the future.

Genetic associations with prostate cancer vary widely with geography and ethnicity, and it is suspected that specific polymorphisms that exist within populations of African American men contribute to poor outcomes. Examination of prostate cancer in African contexts may provide insight into the observations made within African American cohorts and may help explain the increasing incidence of prostate cancer in Africans that results in significant mortality. Future studies should investigate the genetics of prostate cancer in African patients.

Many projects will be necessary to decrease the prostate cancer burden in sub-Saharan Africa such as increasing access to health care, earlier referral to cancer institutes, development of radiotherapy centers, and development of programs to increase continuity of care. These clinical efforts combined with addition research on prostate cancer in sub-Saharan Africa, including epidemiologic, genetic, and clinical trials, are needed to better understand and to improve the prognosis for men with prostate cancer.
